# Heart rate variability as a preictal marker for determining the laterality of seizure onset zone in frontal lobe epilepsy

**DOI:** 10.3389/fnins.2024.1373837

**Published:** 2024-05-09

**Authors:** Seho Lee, Hayom Kim, Jin Hyung Kim, Mingyeong So, Jung Bin Kim, Dong-Joo Kim

**Affiliations:** ^1^Department of Brain and Cognitive Engineering, Korea University, Seoul, Republic of Korea; ^2^Department of Artificial Intelligence, Korea University, Seoul, Republic of Korea; ^3^Department of Neurology, Korea University Anam Hospital, Korea University College of Medicine, Seoul, Republic of Korea; ^4^NeuroTx, Co., Ltd., Seoul, Republic of Korea

**Keywords:** frontal lobe epilepsy, heart rate variability, laterality, seizure onset zone, autonomic nervous system

## Abstract

Determining the laterality of the seizure onset zone is challenging in frontal lobe epilepsy (FLE) due to the rapid propagation of epileptic discharges to the contralateral hemisphere. There is hemispheric lateralization of autonomic control, and heart rate is modulated by interactions between the sympathetic and parasympathetic nervous systems. Based on this notion, the laterality of seizure foci in FLE might be determined using heart rate variability (HRV) parameters. We explored preictal markers for differentiating the laterality of seizure foci in FLE using HRV parameters. Twelve patients with FLE (6 right FLE and 6 left FLE) were included in the analyzes. A total of 551 (460 left FLE and 91 right FLE) 1-min epoch electrocardiography data were used for HRV analysis. We found that most HRV parameters differed between the left and right FLE groups. Among the machine learning algorithms applied in this study, the light gradient boosting machine was the most accurate, with an AUC value of 0.983 and a classification accuracy of 0.961. Our findings suggest that HRV parameter-based laterality determination models can be convenient and effective tools in clinical settings. Considering that heart rate can be easily measured in real time with a wearable device, our proposed method can be applied to a closed-loop device as a real-time monitoring tool for determining the side of stimulation.

## 1 Introduction

Frontal lobe epilepsy (FLE) is the second most common subtype of focal onset epilepsy, and ~30 % of patients with FLE develop drug resistance, making them candidates for surgical treatment or non-invasive neuromodulation (Baumgartner et al., 1997). Although demonstrating the laterality of the seizure onset zone is a fundamental step in presurgical evaluation (Jobst et al., 2000; Miller and Fine, 2022) and in mapping for targeted stimulation (Fisher and Velasco, 2014), it is challenging to determine it in FLE due to the rapid propagation of epileptic discharges to the contralateral hemisphere through the corpus callosum and widespread connectivities (Matsumoto et al., 2007; Chu et al., 2017). Electroencephalography (EEG) data acquisitions through long-term monitoring and high-density channels have been applied for lateralization and localization of precise seizure foci (Lantz et al., 2003), but the examination process is complicated and requires a considerable time and economic burden. Therefore, surrogate markers derived from data that are convenient to acquire and can be processed quickly would have clinical utility in determining the laterality of seizure foci in FLE.

It is well-known that there is hemispheric lateralization of autonomic control (Oppenheimer et al., 1992; Yoon et al., 1997; Kim et al., 2014, 2021; Phillips et al., 2020), and that heart rate (HR) is modulated by interactions between the sympathetic and parasympathetic nervous systems. Based on this knowledge, heart rate variability (HRV) has been widely used to determine the hemispheric lateralization of autonomic control (Rajendra Acharya et al., 2006). Indeed, differences in HRV changes according to hemispheric laterality were demonstrated during unilateral amobarbital injection, suppressing the ipsilateral brain function, in patients with temporal lobe epilepsy (TLE; Yoon et al., 1997). Specifically, sympathetic activation was derived from left hemisphere injection, and parasympathetic activation was derived from right hemisphere injection (Yoon et al., 1997). The capability of HRV parameters for determining the laterality of seizure foci was reaffirmed in TLE (Dono et al., 2020; Sivathamboo and Perucca, 2021). Since the various frontal cortices (e.g., anterior cingulate cortex) are known as fundamental components of the central autonomic network (CAN; Benarroch, 1993), as well as structures in the temporal lobe (e.g., insular cortex), the lateralization of seizure foci in FLE might also be determined using HRV parameters. Considering that recent technological advances in digital health system have allowed the heart rate (HR) to be monitored reliably and conveniently using wearable devices (e.g., smart watch; Lee et al., 2018), HR-based markers for determining the laterality of seizure foci in FLE may be useful in closed-loop neuromodulation systems (Supplementary Figure 1). To the best of our knowledge, no study has investigated the applicability of HRV parameters to determine the laterality of seizure foci in FLE.

Herein, we aimed to compare HRV parameters between right and left FLE. In addition, if there were differences in HRV parameters, we sought to evaluate the applicability of HRV parameters as surrogate markers for determining the laterality of seizure foci in FLE using machine learning algorithms. We hypothesized that HRV parameters reflecting sympathovagal balance might shift toward sympathetic dominance in right FLE and parasympathetic dominance in left FLE. We also hypothesized that HRV parameter-based machine learning models might accurately classify the laterality of seizure foci in FLE.

## 2 Materials and methods

### 2.1 Subjects and data acquisition

This study was based on a retrospective review of an inpatient long-term video-EEG monitoring database at Korea University Anam Hospital between January 2019 and December 2020. From the entire database, we first screened patients with EEG data containing ictal discharges and selected patients confirmed to have FLE through a comprehensive evaluation, including neurological examination, electroclinical diagnosis by board-certified epileptologists (HK, JBK), and neuroimaging studies. All participants had epilepsy with no underlying structural lesions, except for transient seizure-related signal changes. Only FLE patients having EEG data with two or more ictal discharges originating in the frontal lobe of entirely consistent laterality were included in this study, and when seizure activity occurred repeatedly at intervals of <10 min, to such an extent that the timing of ictal onset and termination was unclear, were excluded. The criteria for determining the laterality of the seizure onset zone were established when all of the following conditions were met: (1) the presence of seizure semiology that clearly suggests laterality; (2) demonstration of at least two ictal EEGs compatible with the seizure semiology; and (3) the laterality of the seizure onset zone in electrical source imaging consistent with both clinical and electrophysiological findings. EEG recordings were conducted using a 32-channel recording system (Comet-PLUS, Grass Technologies Inc., West Warwick, RI, USA) with electrodes placed according to the International 10–20 system. EEG data were sampled at 200 Hz, and the bandpass filter was set between 0.1 and 70 Hz. The study followed the ethical guidelines of the Declaration of Helsinki and was approved by the Institutional Review Board of Korea University Anam Hospital (No. 2020AN0435).

### 2.2 Exact Low-Resolution Brain Electromagnetic Tomography

**Figure 1:**
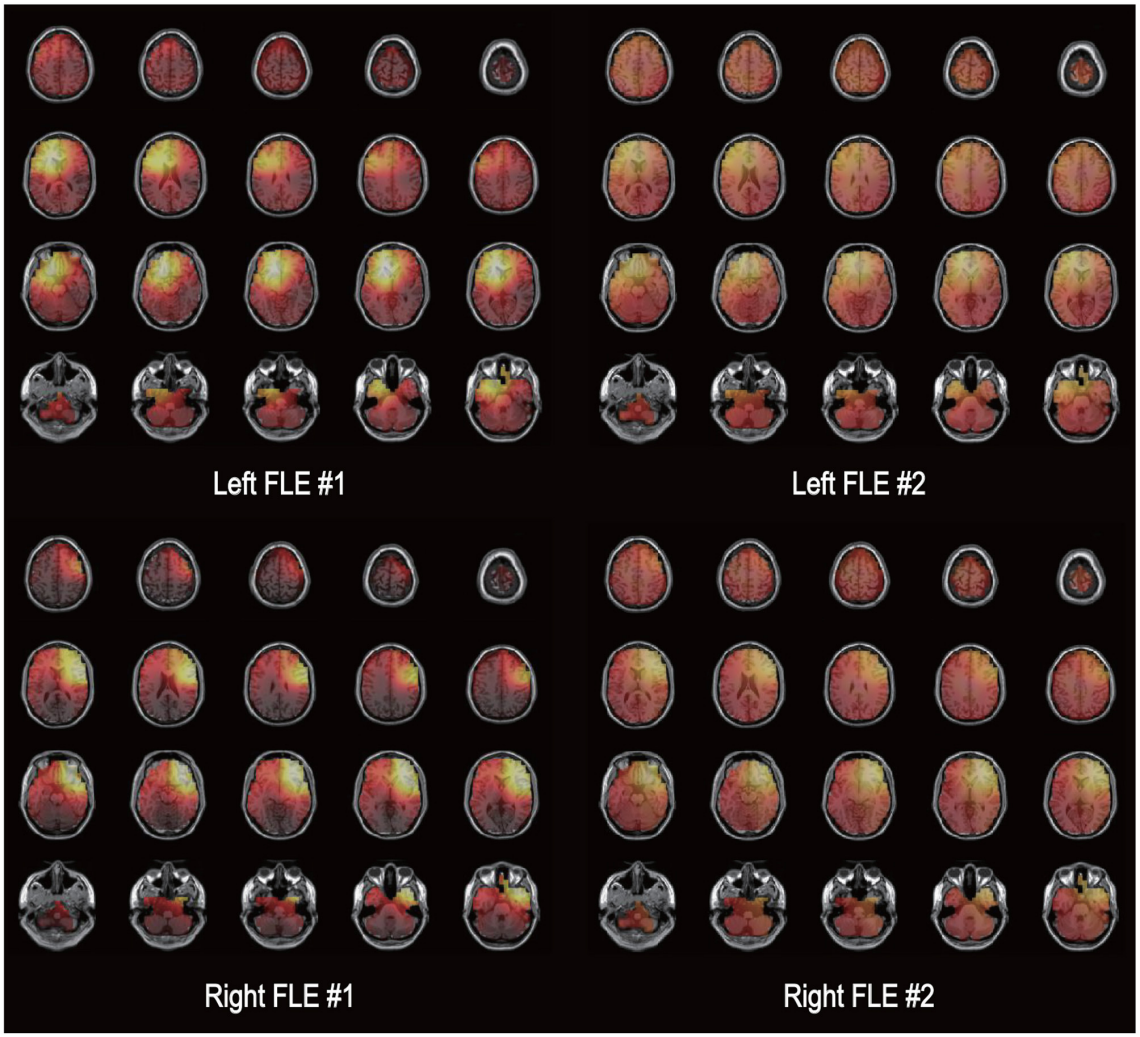
Electrical source imaging. An illustration of the exact low-resolution brain electromagnetic tomography (eLORETA) solutions is shown for two patients with left frontal lobe epilepsy [FLE **(upper row)**] and two patients with right FLE. The source current density of electroencephalography data during 1 min immediately before seizure onset is presented as a bright color. The left side of each image represents the left side of the brain.

Exact Low-Resolution Brain Electromagnetic Tomography (eLORETA) was developed to minimize source localization errors by estimating deeper source locations from standard LORETA (Pascual-Marqui et al., 2011; Aung et al., 2022). eLORETA was calculated to solve the three-dimensional (3D) linear solutions in a three-shell spherical head model, which includes the scalp, skull, and brain tissues. The LORETA solution consists of the voxel current density with estimable power spectral density from EEG on the scalp. eLORETA is a reference-free EEG analysis method that calculates the equal source distribution from independent EEG data. These methods estimate cortical sources that reflect the synchronization of synaptic neural currents associated with local field potentials. To reconfirm the laterality of seizure onset zone, electrical source imaging analysis was performed using EEG data for 1 min immediately before seizure onset (Figure 1; Kovac et al., 2014). In this study, eLORETA analysis was conducted utilizing the Head Atlas Model and the Boundary Element Method (BEM) template for enhanced standardization and comparability across subjects. A symmetric matrix is used as a parameterization for the family of linear imaging methods, where the symmetric matrix is denoted as C and ĵ_*i*_ is an estimator of neuronal activation at the ith voxel. By considering the actual source as an arbitrary point test source at each voxel, it is possible to refine the localization capability of the linear imaging method, where *K*_*j*_ is the lead field matrix and A is a vector containing information of dipole moments for a source. An estimator of neuronal activation can be calculated using the following (Equation 1):


(1) $${\hat{j}}_{i}=\left[{\left(K_{i}^{T}CK_{i}\right)}^{-1/2})K_{i}^{T}C\right]\varphi ,\varphi =k_{j}A$$

These equations can be used to write estimation values as (Equation 2):


(2) $$\begin{array}{l} ||{Ĵ}_{i}|{|}^{2}=A^{T}K_{j}^{T}CK_{i}{\left(K_{i}^{T}CK_{i}\right)}^{+}K_{i}^{T}CK_{j}A \end{array}$$

With regard to eLORETA, we can obtain the density estimate at the ith voxel using following (Equation 3):


(3) $$\begin{array}{l} {ĵ}_{i}=W_{i}^{-1}K_{i}^{T}{\left(KW^{-1}K^{T}+\alpha H\right)}^{+}\varphi \end{array}$$

With the equations given above, exact, zero error localization is possible by selecting the weights derived from (Equation 4):


(4) $$\begin{array}{l} W_{i}={\left[K_{i}^{T}{\left(KW^{-1}K^{T}+\alpha H\right)}^{+}K_{i}\right]}^{1/2} \end{array}$$

### 2.3 Electrocardiography recording and HRV analysis

The ECG signal was simultaneously recorded with the EEG, at a sampling rate of 200 Hz. The single-channel ECG data were extracted during the EEG recording, visually inspected for accuracy and quality, and used for HRV analysis. Ectopic beats and artifacts were discarded, and only normal-to-normal beats were selected for analysis (Camm et al., 1996). Since HR changes dynamically during the transition to ictal onset, it may be appropriate to use HRV parameters analyzed from HR data immediately before ictal onset to accurately determine the laterality of seizure onset zone (Jirsa et al., 2014); herefore, time and frequency domain HRV parameters were calculated using ECG data from 1 min immediately before seizure onset and compared between the left and right FLE groups (Pecchia et al., 2018; Moya-Ramon et al., 2022). The time-domain HRV parameters used in this study are as follows (Camm et al., 1996): mean NN interval (interbeat intervals with artifacts removed, Equation 4), SDNN (standard deviation of NN intervals, Equation 5), SDSD (standard deviation of successive NN interval differences, Equation 6), RMSSD (root mean square of successive NN interval differences, Equation 7), and pNNx (percentage of successive intervals differing by more than x ms, Equation 8).


(5) $$Mean\;NN\;interval(ms)=\frac{1}{n-1}\sum_{i=1}^{n-1}RR_{i+1}-RR_{i}$$

(6) $$SDNN(ms)=\sqrt{\frac{1}{n-1}\sum_{i=1}^{n}{\left(RR_{i}-RR_{mean}\right)}^{2}}$$

(7) $$\begin{array}{l} SDSD(ms)=\\ \sqrt{\frac{1}{n-1}\sum_{i=1}^{n}\left(RR_{i}^{2}-RR_{mean}^{2}\right)-\frac{1}{n-1}\sum_{i=1}^{n}{\left(RR_{i}-RR_{mean}\right)}^{2}} \end{array}$$

(8) $$RMSSD(ms)=\sqrt{\frac{1}{n-1}\sum_{i=1}^{n-1}{\left(RR_{i+1}-RR_{i}\right)}^{2}}$$

(9) $$pNNx=\frac{NNx}{n-1}$$

where x in pNNx indicates threshold of difference between adjacent NN intervals.

The frequency domain of HRV parameters was estimated from the power spectral density (PSD) for NN interval series. The three main frequency component was calculated: the very low frequency (VLF) components under 0.04 Hz, the low frequency (LF) components between 0.04 and 0.15 Hz, and the high frequency (HF) between 0.15 and 0.4 Hz. For estimate the balance of sympathetic tone and parasympathetic tone, the LF/HF ratio was calculated. For estimate the balance of sympathetic tone and parasympathetic tone, the LF/HF ratio was calculated.

### 2.4 Machine learning and statistical analysis

The AutoML technique was implemented for applying various classifiers to determine the laterality of seizure onset in FLE using HRV parameters as input features (He et al., 2021; Qi et al., 2022). Four frequency domain (i.e., VLF, LF, HF, and LF/HF ratio) and six time domain HRV parameters (i.e., mean NN interval, SDNN, RMSSD, SDSD, pNN20, and pNN50) were utilized as input features. Since the range of values in each HRV parameter differs, z-scaled normalized values were applied in the machine learning. The validation of the machine learning classification was conducted through an automated, random, and unbiased selection process. This process utilized a stratified fivefold cross-validation technique to ensure a balanced and representative division between the training and test sets, thereby minimizing potential biases and enhancing the reliability of the results. Specifically, 551 epochs of HRV parameters were randomly divided into five groups for cross-validation. Four groups were assigned to the learning set, and one group to the test set to validate classification accuracy. The same process was repeated five times, and the average value was reported as the final classification performance. The models were learned using optimal parameters selected through grid search analysis. The performance of each classifier was evaluated using a confusion matrix containing the parameters of precision, recall, and accuracy. The recall and specificity were used to generate a receiver operating characteristic (ROC) curve. The area under the ROC curve (AUC) was also calculated. The Shapley Additive Explanations (SHAP) method was applied to the classifier models, showing the most accurate performance in evaluating and ranking the contribution of each variable to the model (Lundberg and Lee, 2017). Group comparisons of demographic and clinical variables were performed using Mann-Whitney *U*-test and Fisher's exact test, where appropriate. HRV parameters were compared between groups using an independent *t*-test. Statistical significance was set to *p* < 0.05. Statistical analyzes were performed with the Statistical Package for the Social Sciences software (Version 25.0; IBM Corp., Armonk, New York, USA).

## 3 Results

### 3.1 Demographic and clinical characteristics

**Figure 2:**
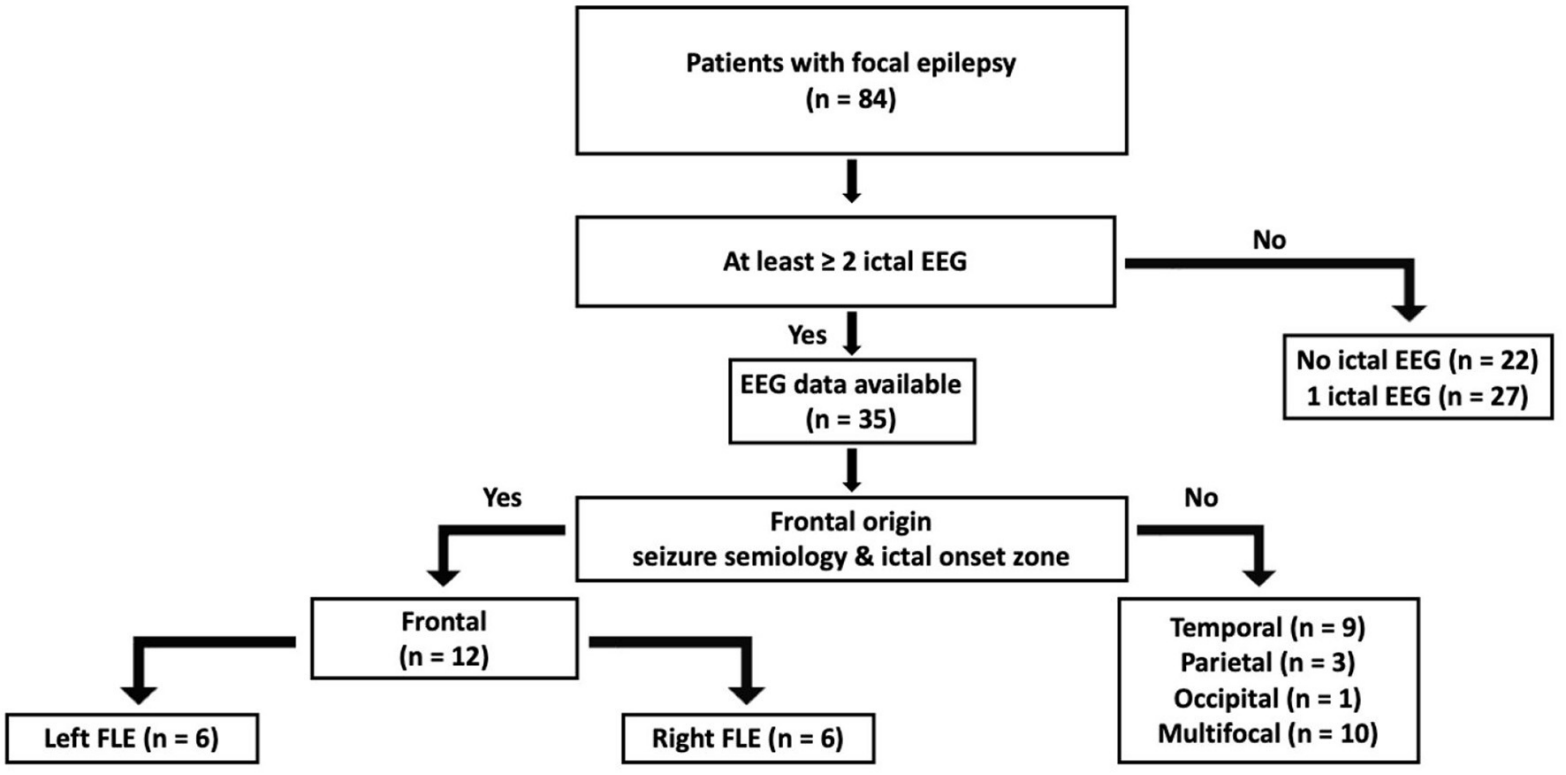
A flow chart of participant classification.

A flow chart of participant selection is presented in Figure 2. Among the 84 patients with focal epilepsy in the database, EEG data with two or more ictal discharges from 35 patients were initially screened in this study. Twenty-three patients having seizure onset zone other than frontal lobe (19 temporal, three parietal, one occipital, and 10 multifocal) were excluded. A total of 12 patients with FLE (6 right and 6 left) were included in the analyzes. A total of 551 (460 left FLE and 91 right FLE) 1 min epoch ECG data were used for HRV analysis. The laterality of seizure onset zone determined by eLORETA was in accordance with that on the basis of electroclinical diagnosis in all patients with FLE. There were no differences in age, age of seizure onset, sex, and proportion of having comorbidities, including hypertension, cardiac arrhythmia, diabetes, and dyslipidemia, between the left and right FLE groups (Table 1).

**Table 1:** Between group comparisons.

**Characteristic**	**Left FLE (460 epochs)**	**Right FLE (91 epochs)**	***p*-value**
**Demographic and clinical variables**			
Age (median, IQR)	62.50 (46.7–70.0)	60.00 (57.5–72.3)	NS
Age of seizure onset (median, IQR)	57.50 (26.3–70.0)	60.00 (57.5–72.3)	NS
Sex (women, %)	50.00	16.67	NS
Hypertension (%)	33.33	50.00	NS
Cardiac arrhythmia (%)	0.00	0.00	NS
Diabetes mellitus (%)	0.00	33.33	NS
Dyslipidemia (%)	0.00	16.67	NS
**Heart rate variability**			
**Frequency domain**			
VLF (ms^2^)	15.14 ± 12.45	25.93 ± 17.13	<0.001
LF (ms^2^)	53.09 ± 17.68	47.63 ± 18.69	<0.001
HF (ms^2^)	31.77 ± 23.00	26.44 ± 25.38	0.048
LF/HF ratio	4.46 ± 7.19	10.18 ± 20.47	<0.001
**Time domain**			
Mean NN interval (ms)	627.35 ± 245.43	629.70 ± 173.45	NS
SDNN (ms)	19.2 ± 13.08	35.55 ± 36.68	<0.001
RMSSD (ms)	11.98 ± 12.70	23.2 ± 30.65	<0.001
SDSD (ms)	8.03 ± 10.675	15.73 ± 21.575	<0.001
pNN20 (%)	0.57 ± 3.85	7.88 ± 16.95	<0.001
pNN50 (%)	0.24 ± 2.33	1.84 ± 5.57	<0.001

FLE, frontal lobe epilepsy; HF, high frequency; IQR, interquartile range; LF, low frequency; NN, normal beat-to-beat; NS, not significant; RMSSD, root mean square of the successive differences; SDNN, standard deviation of NN interval; SDSD, standard deviation of successive RR interval differences; VLF, very low frequency.

### 3.2 HRV parameters

Details of the HRV parameters and statistical results are presented in Table 1. Among the frequency domain HRV parameters, LF and HF power were greater, while VLF and LF/HF ratio were lesser in the left FLE group than in the right FLE group. Compared to the left FLE group, all time domain HRV parameters (i.e., SDNN, RMSSD, SDSD, pNN20, and pNN50), except the mean NN interval, were greater in the right FLE group.

### 3.3 Machine learning

**Figure 3:**
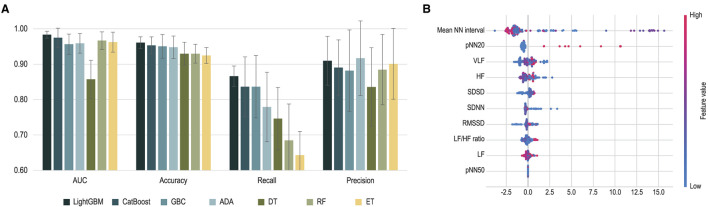
Performance of machine learning classification. **(A)** Performance of each machine learning algorithm is presented. Error bars indicate standard deviations of 5-fold cross-validation. **(B)** Shapley Additive Explanations (SHAP) values extracted from light gradient boosting machines are presented. ADA, adaptive boosting; AUC, area under the curve; DT, decision tree; ET, extra trees; GBC, gradient boosting classifier; HF, high frequency; LF, low frequency; LightGBM, light gradient boosting machine; NN, normal beat-to-beat; RMSSD, root mean square of the successive differences; SDNN, standard deviation of NN interval; SDSD, standard deviation of successive RR interval differences; VLF, very low frequency.

The performance of machine learning algorithms is presented in Figure 3 and Table 2. Among the algorithms for classification, the light gradient boosting machine was the most accurate, with an AUC value of 0.983 and classification accuracy of 0.961. Among the algorithms applied in AutoML methods, CatBoost, gradient boosting classifier, adaptive boost, decision tree, random forest, and extra trees also achieved a high classification accuracy of more than 0.900.

**Table 2:** Details in performance of machine learning classification.

**Model**	**AUC**	**Accuracy**	**Recall**	**Precision**
Light gradient boosting machine	0.983 (0.964)	0.961 (0.940)	0.866 (0.849)	0.910 (0.784)
CatBoost	0.975 (0.969)	0.953 (0.958)	0.836 (0.867)	0.891 (0.872)
Gradient boosting classifier	0.956 (0.967)	0.950 (0.951)	0.836 (0.832)	0.882 (0.859)
Adaptive boost	0.959 (0.918)	0.948 (0.940)	0.779 (0.849)	0.917 (0.789)
Decision tree	0.857 (0.886)	0.930 (0.935)	0.746 (0.815)	0.835 (0.802)
Random forest	0.967 (0.963)	0.930 (0.940)	0.685 (0.780)	0.885 (0.847)
Extra trees	0.962 (0.945)	0.925 (0.935)	0.643 (0.727)	0.901 (0.830)

Values present performance using overall parameters per se [460 epochs in left frontal lobe epilepsy (FLE) vs. 91 epochs in right FLE]; values in parentheses indicate the performance of each classifier after upsampling (460 epochs in left FLE vs. 460 epochs in right FLE).

## 4 Discussion

In this study, we compared HRV parameters derived from preictal period data to investigate the applicability of the HRV parameters to classify the laterality of seizure foci in patients with FLE. We found that most HRV parameters differed between the left and right FLE groups. The groups were classified with a high accuracy using machine learning algorithms with the HRV parameters as input features.

Since the neural activity in the seizure focus is enhanced during the transition from the interictal to the ictal period in epilepsy (Jirsa et al., 2014), sympathetic and parasympathetic activities during the preictal period would be increased in epilepsy with seizure focus on the right and left, respectively. Considering the presence of hemispheric laterality in the modulation of the sympathetic and parasympathetic activities (Oppenheimer et al., 1992; Yoon et al., 1997; Kim et al., 2014, 2021; Phillips et al., 2020), HRV parameters reflecting sympathovagal balance (Rajendra Acharya et al., 2006) can be surrogate markers to determine the laterality of seizure foci. Based on the aforementioned notion, our findings of increased LF/HF ratio and decreased HF power in the right FLE group relative to the left FLE group suggest that FLE patients with seizure foci on the right had increased sympathetic activity during the preictal period and those in the left FLE group suggest the opposite.

There are several lines of evidence indicating that frequency domain HRV parameters could be useful surrogate markers for determining the laterality of seizure foci in TLE (Yoon et al., 1997; Dono et al., 2020). Our findings of the tendency of HRV parameters according to the laterality of seizure foci in FLE were consistent with those in TLE reported previously. CAN is composed of a wide range of areas and is known to regulate autonomic function through functional connectivity between major components such as the brainstem, the insular cortex of the temporal lobe, and the anterior cingulate cortex of the frontal lobe (Benarroch, 1993). Our results suggest that in the context of autonomic function control through CAN, determining seizure foci laterality in FLE patients is possible through HRV parameters, as in TLE.

Indeed, several machine learning algorithms using HRV parameters acquired during the preictal period without other clinical information could classify the laterality of seizure foci with a high accuracy of over 0.900. These findings imply that HRV parameters may be useful surrogate markers for discriminating the laterality in FLE. Given that the ECG data acquisition period was short (i.e., 1 min), HRV parameter-based laterality determination can be a convenient and effective tool in a clinical setting.

The present study had several limitations. First, the sample sizes in the left and right FLE groups were unbalanced, with a relatively small number of epilepsy patients analyzed. Therefore, to avoid biased classification performance, we applied an up-sampling technique, which is one of the methods developed and widely used to minimize the impact of results due to imbalance problems. We found that high classification accuracy, over 0.900, was preserved in all algorithms using up-sampled data; therefore, we believe our results were unlikely to be affected by the data imbalance issue. Second, we could not externally validate the accuracy of machine learning models. Therefore, the generalizability of the accuracy of the differential diagnosis between left and right FLE might be limited, although internal validation has been statistically performed. Third, since frontal lobe encompasses a wide range of regions, the impact on autonomic function can vary by seizure onset zone within specific region of frontal lobe. Further studies in large populations could verify the applicability of determining the laterality of seizure onset zone using HRV parameters according to regions within the frontal lobe. Finally, HRV parameters derived from 1 min recordings may be limited in their ability to serve as a sufficient indicator of autonomous function. However, there are several lines of evidence that HRV parameters derived from short-term recordings were not different from those from 5 min recordings (Pecchia et al., 2018; Moya-Ramon et al., 2022). Therefore, we considered that HRV parameters, derived from 1 min in our study, could serve as appropriate indices for timely forecasting the laterality of seizure onset during real-time monitoring. Moreover, integrated pattern of multiple HRV parameters would be more valuable as a predictor than the independent contribution of individual HRV parameters.

## 5 Conclusion

Using HRV parameters, we explored surrogate markers for differentiating the laterality of seizure foci in FLE patients. Our findings show that machine learning algorithms using HRV parameters could determine the laterality of seizure foci in FLE with a high accuracy of more than 90%. Our results provide insights into the differentiating autonomic states during the preictal period according to the laterality of the seizure onset zone in FLE patients, and HRV parameter-based laterality determination models can be convenient and effective tools in the clinical setting. Furthermore, given the recent advances in application of wearable devices using photoplethysmography signals for healthcare systems (Loh et al., 2022), the laterality of the seizure onset zone in FLE could be determined by using wearable or simple portable devices.

## Data availability statement

The datasets presented in this article are not readily available because of ethical restrictions on the sharing of participant data. Requests to access the datasets should be directed to JK; kjbin80@korea.ac.kr or brainbin80@gmail.com.

## Ethics statement

The studies involving humans were approved by Institutional Review Board of Korea University Anam Hospital (No. 2020AN0435). The studies were conducted in accordance with the local legislation and institutional requirements. The participants provided their written informed consent to participate in this study.

## Author contributions

SL: Writing - original draft, Writing - review & editing, Data curation, Formal analysis, Investigation, Methodology, Software, Visualization. HK: Conceptualization, Data curation, Formal analysis, Investigation, Methodology, Project administration, Resources, Writing - original draft, Writing - review & editing. JHK: Data curation, Formal analysis, Methodology, Supervision, Validation, Writing - original draft, Writing - review & editing. MS: Conceptualization, Data curation, Supervision, Validation, Visualization, Writing - original draft, Writing - review & editing. JBK: Conceptualization, Data curation, Formal analysis, Funding acquisition, Investigation, Methodology, Project administration, Resources, Software, Supervision, Validation, Visualization, Writing - original draft, Writing - review & editing. D-JK: Conceptualization, Formal analysis, Funding acquisition, Methodology, Project administration, Resources, Supervision, Validation, Writing - original draft, Writing - review & editing.
