# Supplementary material for: Heart rate variability as a preictal marker for determining the laterality of seizure onset zone in frontal lobe epilepsy
Source: Front Neurosci. 2024 May 9;18:1373837. doi: 10.3389/fnins.2024.1373837 (PMC11114103; doi:10.3389/fnins.2024.1373837)
Supplement: Supplementary file 1 [file Image_1.pdf]

Supplementary Material

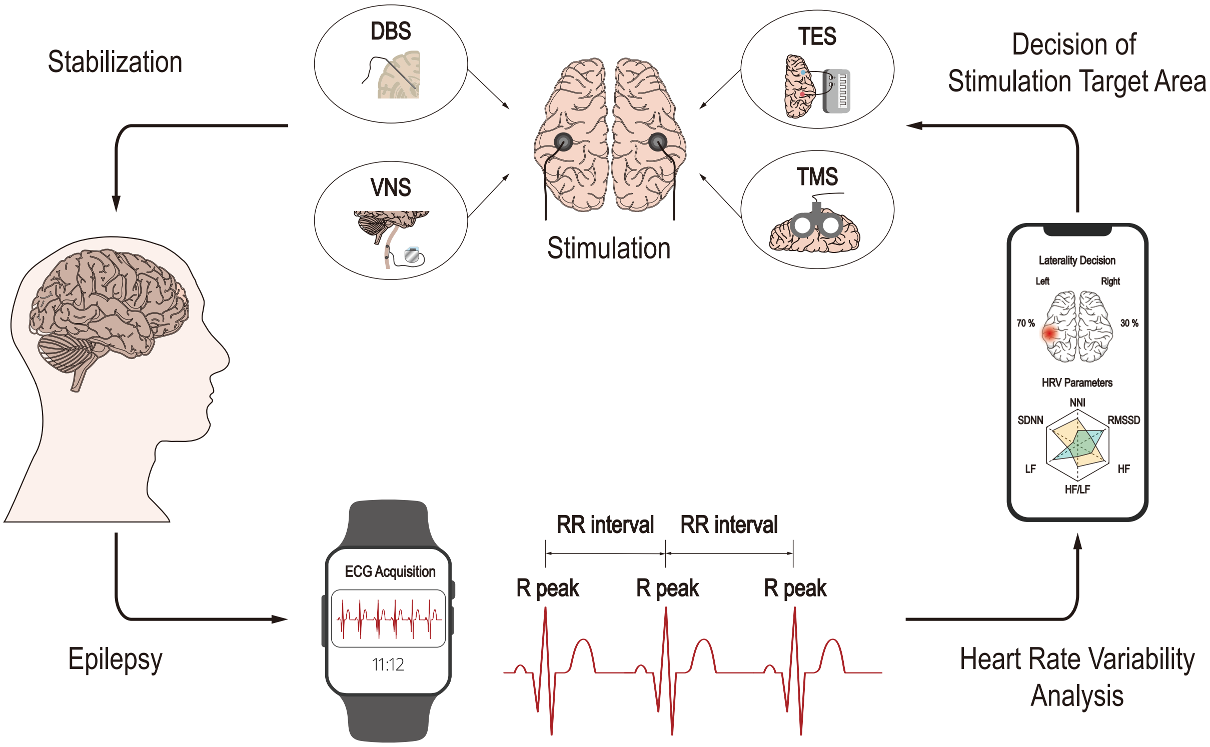

Figure 1. Conceptual diagram: applicability of this research. The figure shows an overview of a neuromodulation system for epilepsy patients that uses electrocardiograms acquired from wearable devices to determine the laterality of epileptic foci and then targets the stimulation to the side of the brain where the epileptic foci are located. ECG, electrocardiography, DBS, deep brain stimulation, VNS, vagus nerve stimulation, TES, transcranial electric stimulation, TMS, transcranial magnetic stimulation.
